# Plasmin Generation Potential and Recanalization in Acute Ischaemic Stroke; an Observational Cohort Study of Stroke Biobank Samples

**DOI:** 10.3389/fneur.2020.589628

**Published:** 2020-11-03

**Authors:** Thomas Lillicrap, Charithani B. Keragala, Dominik F. Draxler, Jilly Chan, Heidi Ho, Stevi Harman, Be'eri Niego, Elizabeth Holliday, Christopher R. Levi, Carlos Garcia-Esperon, Neil Spratt, Prajwal Gyawali, Andrew Bivard, Mark W. Parsons, Joan Montaner, Alejandro Bustamante, Israel Fernandez Cadenas, Geoffrey Cloud, Jane M. Maguire, Lisa Lincz, Timothy Kleinig, John Attia, Simon Koblar, Monica Anne Hamilton-Bruce, Philip Choi, Bradford B. Worrall, Robert L. Medcalf

**Affiliations:** ^1^Department of Neurology, John Hunter Hospital, Newcastle, NSW, Australia; ^2^Hunter Medical Research Institute, University of Newcastle, Newcastle, NSW, Australia; ^3^Australian Centre for Blood Diseases, Monash University, Melbourne, VIC, Australia; ^4^Department of Cardiology, University Hospital of Bern, Bern, Switzerland; ^5^Bern Centre for Precision Medicine, Bern, Switzerland; ^6^Sydney Partnership for Health, Education, Research and Enterprise, Sydney, NSW, Australia; ^7^Neurology Department, Royal Melbourne Hospital, Melbourne, VIC, Australia; ^8^School of Medicine, University of New South Wales, Sydney, NSW, Australia; ^9^Neurovascular Research Laboratory, Vall d'Hebron Institute of Research (VHIR), Barcelona, Spain; ^10^Stroke Research Program, Institute of Biomedicine of Seville, IBiS/Hospital Universitario Virgen del Rocío, Consejo Superior de Investigaciones Científicas (Spanish National Research Agency), University of Seville, Seville, Spain; ^11^Department of Neurology, Hospital Universitario Virgen Macarena, Seville, Spain; ^12^Stroke Pharmacogenomics and Genetics Lab, Sant Pau Hospital Institute of Research, Barcelona, Spain; ^13^Department of Neurology, The Alfred Hospital, Melbourne, VIC, Australia; ^14^Department of Clinical Neuroscience, School of Nursing and Midwifery, Central Clinical School, Monash University, Melbourne, VIC, Australia; ^15^Department of Haematology, University of Technology Sydney, Sydney, NSW, Australia; ^16^Haematology Department, Calvary Mater Newcastle, Waratah, NSW, Australia; ^17^Neurology Department, Royal Adelaide Hospital, Adelaide, SA, Australia; ^18^Adelaide Medical School, The University of Adelaide, Adelaide, SA, Australia; ^19^Neurology, Central Adelaide Local Health Network, Adelaide, SA, Australia; ^20^Department of Neurosciences, Eastern Health, Melbourne, VIC, Australia; ^21^Eastern Health Clinical School, Faculty of Medicine, Nursing and Health Sciences, Monash University, Melbourne, VIC, Australia; ^22^Department of Neurology, University of Virginia, Charlottesville, VA, United States; ^23^Department of Public Health Sciences, University of Virginia, Charlottesville, VA, United States

**Keywords:** acute stroke therapy, fibrinolysis, rtPA, thrombolysis, plasmin, stroke, recanalization

## Abstract

**Rationale:** More than half of patients who receive thrombolysis for acute ischaemic stroke fail to recanalize. Elucidating biological factors which predict recanalization could identify therapeutic targets for increasing thrombolysis success.

**Hypothesis:** We hypothesize that individual patient plasmin potential, as measured by *in vitro* response to recombinant tissue-type plasminogen activator (rt-PA), is a biomarker of rt-PA response, and that patients with greater plasmin response are more likely to recanalize early.

**Methods:** This study will use historical samples from the Barcelona Stroke Thrombolysis Biobank, comprised of 350 pre-thrombolysis plasma samples from ischaemic stroke patients who received serial transcranial-Doppler (TCD) measurements before and after thrombolysis. The plasmin potential of each patient will be measured using the level of plasmin-antiplasmin complex (PAP) generated after *in-vitro* addition of rt-PA. Levels of antiplasmin, plasminogen, t-PA activity, and PAI-1 activity will also be determined. Association between plasmin potential variables and time to recanalization [assessed on serial TCD using the thrombolysis in brain ischemia (TIBI) score] will be assessed using Cox proportional hazards models, adjusted for potential confounders.

**Outcomes:** The primary outcome will be time to recanalization detected by TCD (defined as TIBI ≥4). Secondary outcomes will be recanalization within 6-h and recanalization and/or haemorrhagic transformation at 24-h. This analysis will utilize an expanded cohort including ~120 patients from the Targeting Optimal Thrombolysis Outcomes (TOTO) study.

**Discussion:** If association between proteolytic response to rt-PA and recanalization is confirmed, future clinical treatment may customize thrombolytic therapy to maximize outcomes and minimize adverse effects for individual patients.

## Introduction

Despite the increasing use of mechanical thrombectomy (MT), thrombolysis with recombinant tissue-type plasminogen activator (rt-PA) remains a cornerstone of acute ischaemic stroke treatment. Upon administration, rt-PA cleaves endogenous plasminogen (a free-floating but inactive protein) into its active form plasminogen; using fibrin as a cofactor so that the majority of plasmin generation occurs at the sight of a fibrinous clot. Plasmin is rapidly bound by α2-antiplasmin to form the inactive plasmin-antiplasmin (PAP) complex but is a potent fibrinolytic nonetheless (1). However, more than 60% of patients who receive rt-PA fail to recanalize (2) and this failure of recanalization has been associated with poor patient outcomes, even compared to patients who did not receive any reperfusion therapy (3). While great strides have been made with regard to selecting patients who are likely to have a favorable clinical outcome after thrombolysis (4–6), the reasons some patients fail to recanalize remain largely unknown. Previous studies have examined baseline levels of individual fibrinolysis markers in acute ischemic stroke patients (7) but this approach is limited as the potency of thrombolysis depends on the net capacity of a patient's plasma to generate plasmin from plasminogen in response to rt-PA and this may not necessarily correlate with baseline levels of individual fibrinolysis markers. We aim to examine association between net plasmin generation in the presence of rt-PA and clinical outcomes, particularly time to recanalization.

## Methods and Analysis

### Sample Collection

Blood samples were collected via venepuncture into sodium-citrate anticoagulated vacuum-tubes during routine pre-thrombolysis work-up. Extracted plasma was stored at −80°C until analyzed.

### Inclusion and Exclusion Criteria

Samples from patients who received thrombolysis and underwent transcranial doppler (TCD) before thrombolysis, and at least once within 6 h after thrombolysis will be included in the primary analysis. In addition, samples from patients who received thrombolysis and underwent angiography, either computed tomography (CT) or magnetic resonance imaging (MRI), before and 24-h post-thrombolysis will be included in secondary analyses.

### Plasmin Assays (Lab Work)

Pre-thrombolysis plasma samples will be analyzed for both baseline fibrinolytic markers and *ex-vivo* plasmin generating capacity using different modalities.

All samples will be tested for baseline total plasminogen, α2-antiplasmin, plasminogen activator inhibitor-1 (PAI-1) levels, and baseline t-PA activity using specific enzyme-linked immunosorbent assays (ELISA) from Molecular Innovations, USA.

Plasmin-antiplasmin (PAP) complexes will be measured as a surrogate for rt-PA inducible plasmin activity generated after addition of rt-PA. To achieve this, PAP complexes will be measured at baseline and also after the *ex-vivo* addition of 50 nM rt-PA (“Inducible PAP”) in the presence and absence of a soluble fibrin co-factor, Cyanogen bromide activated Fibrinogen (CNBr-Fib, 0.25 mg/ml) Both ELISA kit and CNBr-Fib used for the PAP analysis are from DRG International (USA).

Inducible PAP levels will be measured following stimulation of the plasma samples with vehicle (for baseline levels), addition of CNBr-Fib alone, rt-PA alone, and both rt-PA + CNBr-Fib for 10 min at 37°C and the reaction stopped by combining with aprotinin (20 μM), a serine protease inhibitor which competitively inhibits plasmin. The generated PAP complexes will then be quantified using a commercial ELISA (DRG International, USA). Treatment of samples with cofactor alone is to determine whether plasma is able to generate a significant amount of inducible PAP on its own, due to elevated levels of endogenous t-PA that may occur during acute ischaemic stroke.

Furthermore, the rate of plasmin generation within each sample will also be evaluated with an amidolytic assay using S2251, a chromogenic substrate specific for plasmin (8). The production of plasmin within the sample hydrolyses S-2251, liberating a pNA chromogenic group detected on a plate reader at an absorbance wavelength of 405 nm.

PAP complex levels will also be determined in plasma samples obtained at various time points post-rt-PA thrombolysis (1, 2, 12, and 24 h). Hence the degree of rt-PA inducible PAP complexes determined in the pre-thrombolysis samples will then be correlated to the actual degree of PAP complexes formed *in vivo* after rt-PA administration. This will also provide relevant information as to the temporal nature of the response to rt-PA treatment.

### Imaging/Recanalization Assessment

**Figure 1:**
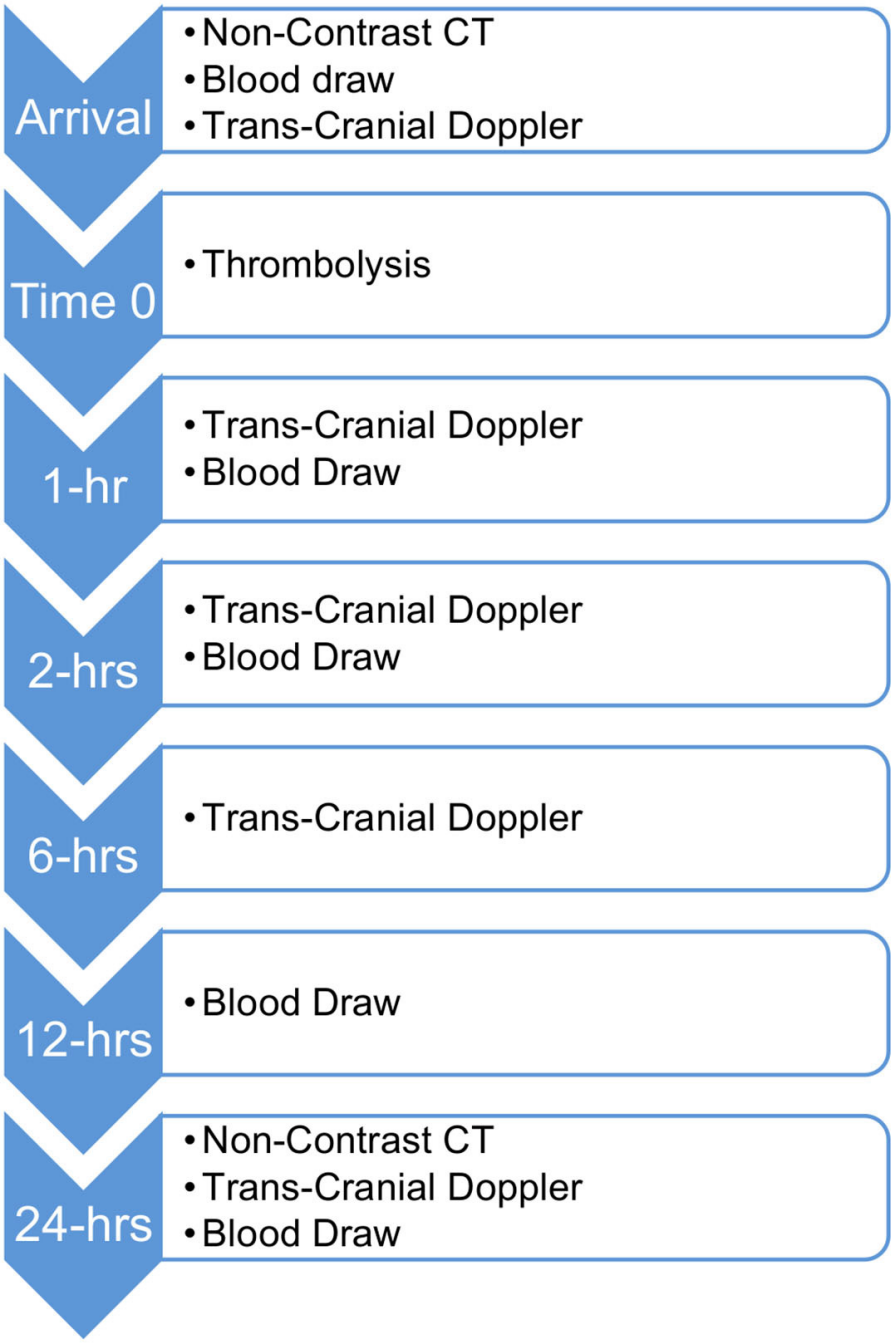
Sample and data collection timeline from patient arrival at hospital.

Patients in the primary analysis received non-contrast computed tomography (NCCT) to rule out hemorrhage and TCD pre-thrombolysis and at 1, 2, 6, and 24-h post-thrombolysis (see Figure 1). The initial occlusion and subsequent recanalization were assessed using the TIBI score. For the purposes of this analysis a TIBI score ≥4 will be considered as recanalization.

### Statistical Analysis

The primary analysis will use Cox proportional hazards regression to estimate the relationship between plasmin potential variables and time to recanalization, defined by a thrombolysis in brain ischemia (TIBI) score of 4 or 5 (9).

The key predictive variables will be the fibrinolytic potential, as measured by the *in vitro* response to thrombolytic agent. This response will be measured in terms of the amount of PAP formed, the ratio of formed PAP to baseline PAP or the rate of plasmin activity. The Kaplan-Meier survival function for various levels of the key predictors will be visualized and a Cox Proportional-Hazard regression will be used to estimate the hazard ratio with 95% confidence intervals for the effect of each key predictor on time to recanalization after adjusting for potential confounders. Potential confounders include blood glucose level, initial occlusion size (as measured by baseline TIBI), age, sex, prior medications, and chronic illness. The proportional hazards assumption will be tested using standard statistical tests and residual plots. Predictive accuracy will be quantified using c-statistics.

Secondary analyses will use logistic regressions to examine the correlations between fibrinolytic markers and recanalization within 6 h and outcome at 24-h post-thrombolysis, recanalization and haemorrhagic transformation of cerebral infarcts detected on follow-up CT and disability at 3 months as measured by the modified Rankin scale.

### Available Sample Size

The Barcelona Stroke Thrombolysis Biobank consists of samples from a total 350 patients who underwent TCD before thrombolysis and NCCT before and 24-h after thrombolysis, 332 of whom underwent TCD at least once after thrombolysis (see Table 1). The TOTO (10) sample is forecast to contain samples from ~250 patients by the time analysis is conducted, ~120 of these patients will have vessel occlusions visible on CTA or MRA.

**Table 1:** Number of patients with Trans-Cranial Doppler at each time point in the Barcelona Biobank.

**Timepoint**	***N***
0	350
1	332
2	300
6	220
24	194

The sample size available for the primary recanalization analysis will consist of the 332 patients from the Barcelona Stroke Thrombolysis Biobank who underwent at least 1 post-thrombolysis TCD (11). Initially 50% of these samples will be analyzed in a pilot study. If a larger sample is required, this pilot study will allow for formal power calculations to determine the final sample size. The sample size for the logistic regression analysis of recanalization at 24-h will be ~450 including Barcelona Stroke Thrombolysis Biobank samples and those from the TOTO study with clear vessel occlusions. Analysis of haemorrhagic transformation will include all 600 samples (350 from the Barcelona Stroke Thrombolysis Biobank and 250 from TOTO).

## Discussion

Individual variation in fibrinolytic potential remain poorly understood, especially with regard to its effects on clinical outcomes after thrombolysis. The Barcelona Stroke Thrombolysis Biobank provides an exceptional sample in which to examine correlations between fibrinolytic markers and recanalization as the use of serial TCD assessments allows direct assessment of early recanalization, and being collected before mechanical thrombectomy was common practice, the biobank consists entirely of patients who received thrombolysis only. Where subsequent biobank studies such as TOTO have collected much more detailed baseline data in the form of multi-modal CT, such examinations cannot be repeated as often as TCD so these studies lack the timing data available from the Barcelona Stroke Thrombolysis Biobank. In addition, the Barcelona Stroke Thrombolysis Biobank recruited patients between 2003 and 2009, before the widespread use of mechanical thrombectomy and was therefore purely focussed on thrombolysis. Subsequent biobanks (including TOTO) have been forced to either recruit patients who undergo mechanical thrombectomy, or recruit fewer patients with large-vessel occlusions.

If individual fibrinolytic potential does correlate with time to recanalization, future studies could focus on reducing variation in fibrinolytic potential, for example by supplementing plasminogen in patients before thrombolysis.

## Ethics Statement

The studies involving human participants were reviewed and approved by the Barcelona Stroke Biobank received ethical approval from Vall d'Hebron Hospital ethics committee [Registration No. PT(HG)89/2003]. The TOTO Biobank Study received ethical approval from the Hunter New-England Health Human Research Ethics Committee (Registration No. 14/10/15/4.02). The study combining samples from these two biobanks was approved by the Monash University Human Research Ethics Committee (Registration No. 21640) with appropriate material transfer agreements between the University of Newcastle and Monash University, and between VHIR and Monash University. The patients/participants provided their written informed consent to participate in this study.

## Author Contributions

RM conceived and oversaw the development of the study. TL and EH wrote the statistical analysis plan. DD, JC, HH, SH, and BN developed the laboratory protocols. TL, EH, RM, CL, CG-E, NS, PG, ABi, MP, GC, JMM, LL, TK, JA, SK, MH-B, BW, and RM all oversee the TOTO biobank and contribute to the design of studies arising from it. JM, ABu, and IC contributed to the management of and access to the Barcelona Stroke Biobank and the design of the study. TL drafted the manuscript. All authors critically reviewed and edited the manuscript and have approved the final draft.

## Conflict of Interest

The authors declare that the research was conducted in the absence of any commercial or financial relationships that could be construed as a potential conflict of interest.
